# Development and validation of a nomogram model for predicting the risk of MAFLD in the young population

**DOI:** 10.1038/s41598-024-60100-y

**Published:** 2024-04-23

**Authors:** Yi Yuan, Muying Xu, Xuefei Zhang, Xiaowei Tang, Yanlang Zhang, Xin Yang, Guodong Xia

**Affiliations:** 1 Department of Gastroenterology, The Affiliated Hospital of Southwest Medical University, Luzhou, 646000 Sichuan China; 2 The People’s Hospital Of Luzhou, Luzhou, 646000 Sichuan China; 3 Department of Health Management Center, The Affiliated Hospital of Southwest Medical University, Luzhou, 646000 Sichuan China

**Keywords:** MAFLD, Young population, Nomogram, Risk prediction, Health care, Pathogenesis, Risk factors

## Abstract

This study aimed to develop and validate a nomogram model that includes clinical and laboratory indicators to predict the risk of metabolic-associated fatty liver disease (MAFLD) in young Chinese individuals. This study retrospectively analyzed a cohort of young population who underwent health examination from November 2018 to December 2021 at The Affiliated Hospital of Southwest Medical University in Luzhou City, Sichuan Province, China. We extracted the clinical and laboratory data of 43,040 subjects and randomized participants into the training and validation groups (7:3). Univariate logistic regression analysis, the least absolute shrinkage and selection operator regression, and multivariate logistic regression models identified significant variables independently associated with MAFLD. The predictive accuracy of the model was analyzed in the training and validation sets using area under the receiver operating characteristic (AUROC), calibration curves, and decision curve analysis. In this study, we identified nine predictors from 31 variables, including age, gender, body mass index, waist-to-hip ratio, alanine aminotransferase, low-density lipoprotein cholesterol, high-density lipoprotein cholesterol, uric acid, and smoking. The AUROC for the subjects in the training and validation groups was 0.874 and 0.875, respectively. The calibration curves show excellent accuracy of the nomogram. This nomogram which was based on demographic characteristics, lifestyle habits, anthropometrics, and laboratory data can visually and individually predict the risk of developing MAFLD. This nomogram is a quick and effective screening tool for assessing the risk of MAFLD in younger populations and identifying individuals at high risk of MAFLD, thereby contributing to the improvement of MAFLD management.

## Introduction

MAFLD refers to a fatty liver disease resulting from systemic metabolic dysregulation, and is renamed from non-alcoholic fatty liver disease (NAFLD). According to the related statistics, the incidence and prevalence of MAFLD are rapidly increasing globally, which grows currently by about 25% worldwide^1^.MAFLD is increasingly becoming a disastrous threat to human health.

Due to improved nutrition and a significant shift in dietary habits among young people, the number of cases of obesity in this age group continues to rise^2^. Meanwhile, the onset of MAFLD is insidious and often asymptomatic in its early stages. Without timely diagnosis and treatment, MAFLD can lead to chronic hepatitis, cirrhosis, and hepatocellular carcinoma. It can also contribute to the progression of other metabolism-related disorders such as diabetes mellitus, hyperlipidemia, and hyperuricemia^3,4^. Neglecting the health status of the younger population may also lead to the continued progression of MAFLD and result in a significant health burden. Therefore, early detection and management are essential to prevent the progression of MAFLD. By far, among all the available diagnostic tools, liver biopsy is regarded as the gold standard for the diagnosis of MAFLD. Nevertheless, due to its invasive nature, liver biopsy is rarely recommended for routine screening. Other imaging techniques, such as magnetic resonance spectroscopy (MRS) and computed tomography (CT) are neither cost-friendly for mass screening nor user-friendly (difficult to generalize). Ultrasound seems to be widely used as the diagnostic modality for identifying steatosis for its cost and convenience. Yet its accuracy heavily relied on the operator’s personal experience, let alone its sensitivity for detection of < 20% steatosis, all these factors have constrained MAFLD screening in developing or impoverished areas^5^. Therefore, it is profound to develop a simple, non-invasive, and practical prediction model for rapid and effective screening of MAFLD. Several simple screening tools for MAFLD have emerged based on demographics, laboratory factors, and anthropometrics^6,7^. However, these models may only focus on the overweight population^8^ or concentrate solely on the medical population in the United States, thus applicability to Chinese is yet determined^9^. Consequently, no suitable MAFLD screening tool has been developed for the young Chinese population.

Nomogram is widely used. It is a graphical prediction tool that visually quantifies an event's risk based on the variables’ specificity^10^. We aimed to develop and validate a nomogram based on routine indicators associated with MAFLD during health examinations, in order to screen for MAFLD in young Chinese populations.

## Materials and methods

### Participants

**Figure 1:**
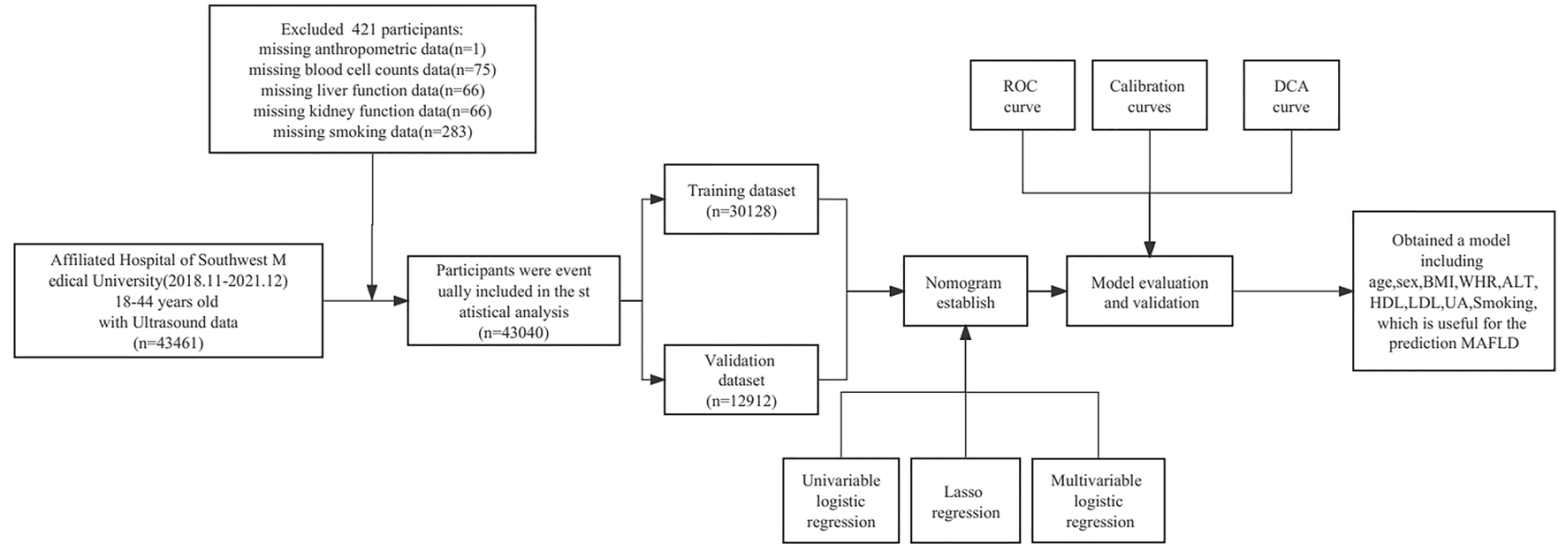
Flow diagram of study design.

This study retrospectively included a population of young adults (18–44 years old) who underwent health examination at The Affiliated Hospital of Southwest Medical University in Sichuan Province, China, from November 2018 to December 2021, including a total of 43,461 subjects with complete ultrasound data examinations. We obtained Participant demographics, anthropometry, laboratory tests, and personal history data from electronic medical examination systems. The research flow chart is shown in Fig. 1. All methods were carried out in accordance with relevant guidelines and regulations. All experimental protocols were approved by Ethics Committee of the Affiliated Hospital of Southwest Medical University. Informed consent was waived by the Ethics Committee of the Affiliated Hospital of Southwest Medical University because of the retrospective nature of our study.

### Data collection

The collected variables included factors that previous studies have demonstrated to be associated with MAFLD, including demographics (age, sex), anthropometrics (body mass index [BMI], systolic blood pressure [SBP], diastolic blood pressure [DBP], waist circumference [WC], waist-to-hip ratio [WHR]), laboratory tests (neutrophil [NEU], lymphocyte [LYM], white blood cell [WBC], red blood cell [RBC], platelets [PLT], hemoglobin [HGB], alanine aminotransferase [ALT], aspartate aminotransferase [AST], alkaline phosphatase [ALP], low-density lipoprotein cholesterol [LDL-C], high-density lipoprotein cholesterol [HDL-C], uric acid [UA], total cholesterol [TC], triglyceride [TG], total protein [TP], globulin [GLO], albumin [ALB], total bilirubin [TBIL], direct bilirubin [DBIL], indirect bilirubin [IBIL], creatinine [Crea], glomerular filtration rate [GFR], fasting blood glucose [FPG]), living habits (Smoking).

Our study used the simplified Modification of Diet in Renal Disease (MDRD) formula that was improved by the Chinese to calculate glomerular filtration rate (GFR): GFR (ml/[min × 1.73 m^2^]) = 186 × (Cr [mg/dl])^−1.154^ × (age)^−0.203^ × (0.742 if female)^11^.

A total of 31 variables were collected.

### Definition and assessment

We classified BMI into four groups according to the World Health Organisation’s BMI standards for Asians: underweight: < 18.50 kg/m^2^,normal: 18.50–22.99 kg/m^2^,overweight: 23.00–24.99 kg/m^2^, obese: ≥ 25.00 kg/m^2^^12^.

After resting for 5 min before the blood pressure measurement, the participant took the right arm blood pressure, including systolic and diastolic blood pressure, in a sitting position. Abnormal systolic blood pressure was defined as systolic blood pressure greater than or equal to 130 mmHg or a previous diagnosis of hypertension, and abnormal diastolic blood pressure was defined as diastolic blood pressure greater than or equal to 85 mmHg or a previous diagnosis of hypertension. We defined WC abnormalities as WC ≥ 90 cm in men and WC ≥ 80 cm in women^3^. Abnormal WHR was defined as ≥ 0.90 in males and ≥ 0.85 in females^13^. Dyslipidemia was defined as follows: TC ≥ 5.2 mmol/L; LDL-C ≥ 3.4 mmol/L; HDL-C < 1 mmol/L in men and < 1.3 mmol/L in women; TG ≥ 1.7 mmol/L^6^. Hyperuricaemia was defined as UA > 420 μmol/L^14^. Our study defined impaired fasting glucose as FPG ≥ 5.6 mmol/L^3^.

### MAFLD

The diagnosis of hepatic steatosis in this study was based on the results of liver ultrasonography. This institution is a tertiary medical center, trained sonographers performed liver ultrasonography, and the ultrasound diagnosis of hepatic steatosis was based on the presence of hepatic and renal echocontrast, liver parenchymal brightness, deep attenuation, and vascular blurring^15^. The diagnosis of MAFLD is based on ultrasound-confirmed hepatic steatosis and one of the following three criteria: overweight or obesity, presence of type 2 diabetes, or metabolic dysfunction. Metabolic dysfunction requires at least two conditions to be met: (1) male WC ≥ 90 cm, female WC ≥ 80 cm; (2) blood pressure ≥ 130/85 mmHg or specific drug treatment; (3) hypertriglyceridemia (TG ≥ 1.70 mmol/L or on lipid-lowering therapy); (4) HDL-C < 1.0 mmol/L in men, < 1.3 mmol/L in women; (5) prediabetes^3^.

### Statistical analysis

For model development, all participants were randomly divided into two groups in a 7:3 ratio for training and validation using the “caret” package. We used the training dataset to develop the model and the validation dataset for internal validation. Then, we assessed the comparability between the two datasets. We selected the t-test or Wilcox rank-sum test for continuous variables based on homogeneity of variances and normality. We selected the chi-squared test or Fisher’s exact test for categorical data (dichotomous and multichotomous) depending on the data condition. Univariate logistic regression analysis was used to identify potential predictors of MAFLD. To ensure that the univariate logistic regression model was not overfitting, LASSO was performed to eliminate factors with high correlation. In subsequent analyses, independent predictors were further identified using multivariate logistic regression. This study then developed a nomogram to predict MAFLD risk using the “rms” package. ROC was used to assess discriminant performance, with an AUROC greater than 0.70 reflecting the high performance of the nomogram. A calibration curve is used to measure the agreement between actual outcomes and predicted probabilities. The clinical utility of the nomogram was assessed by DCA.

Statistical analyses were performed using R software version 4.3.1 and SPSS version 24.0. A two-sided *P* value < 0.05 was considered statistically significant.

### Ethical approval

The study was reviewed by the Ethics Committee of the Affiliated Hospital of Southwest Medical University.

## Results

### Missing values for variables

**Figure 2:**
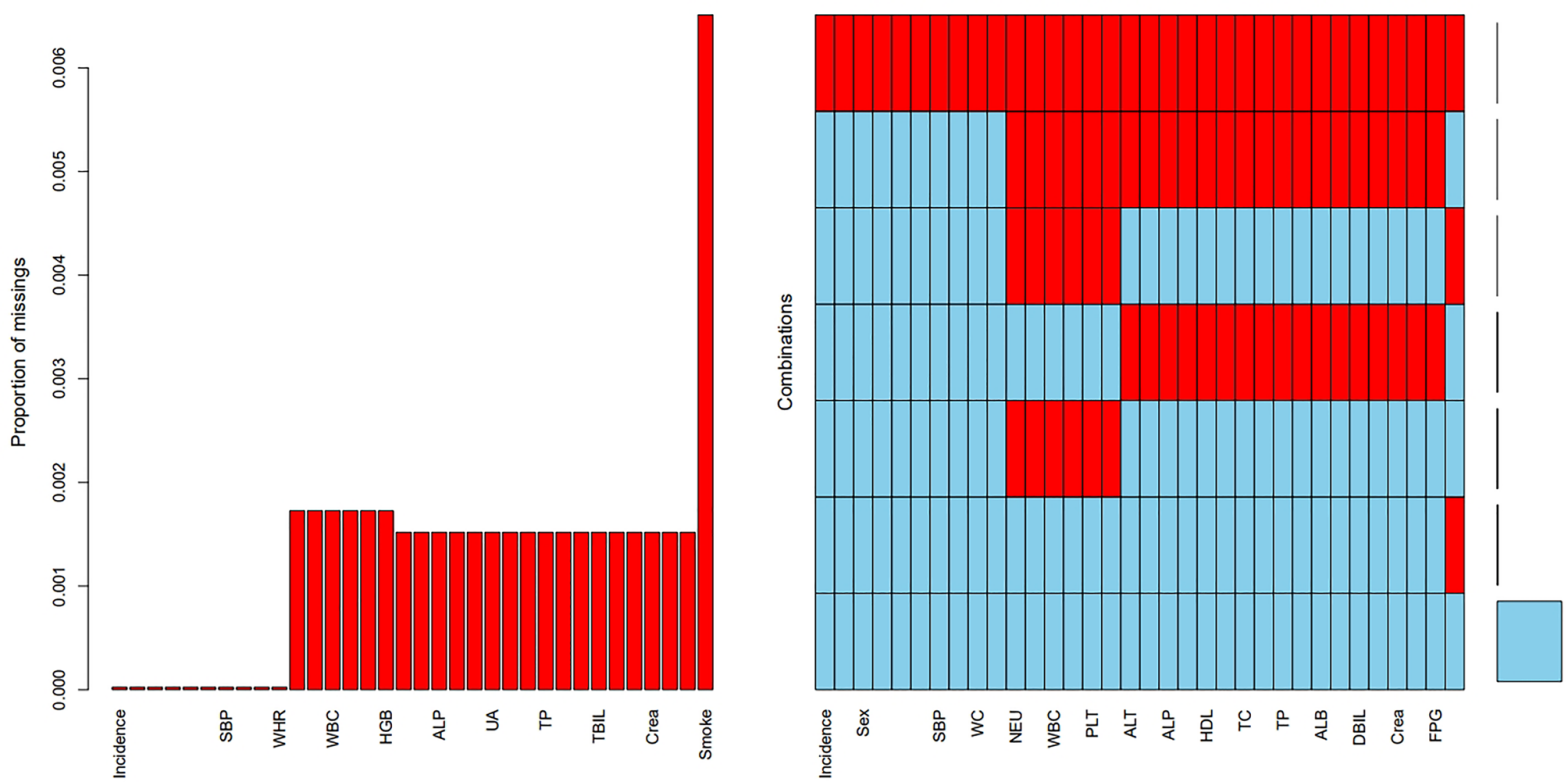
Distribution of missing values for variables.

A missing value test was shown on the collected data, and 1831 missing values were detected. Figure 2 shows missing data for 31 variables. Bar graphs show missing proportions for all variables below 0.6%.

### Study cohorts

Participants were randomly divided into training groups (n = 30,128) and validation groups (n = 12,912) in a ratio of 7:3. There were no statistically significant differences in clinical information or laboratory results between the two cohorts (*P* > 0.05). The characteristics of the two datasets are shown in Supplementary Table 1.

### Selection of major predictors for MAFLD

**Figure 3:**
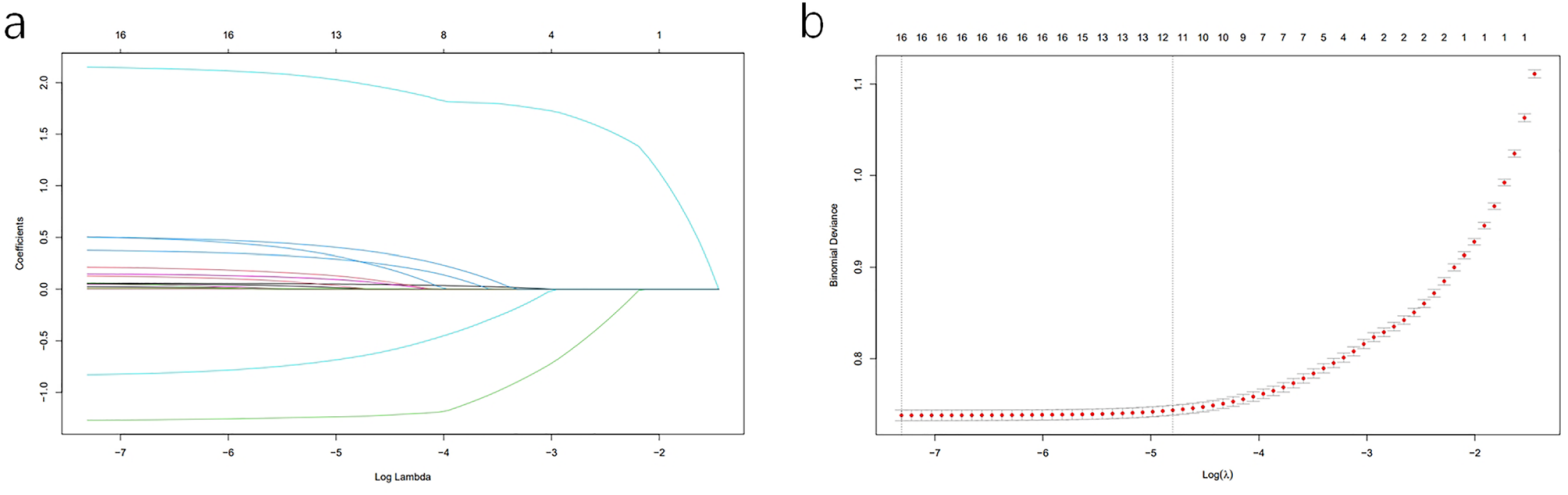
Variable selection via LASSO binary logistic regression model. (**a**) Distribution of LASSO coefficients for 17 variables. (**b**) Select the optimized parameters (lambda) for the LASSO model using ten-fold cross-validation. Plot mean squared error versus log (lambda).

Table 1 shows the results of univariate logistic regression analysis. Univariate logistic regression analysis identified 17 candidate characteristics, including age, gender, BMI, SBP, DBP, WC, WHR, ALT, AST, LDL, HDL, UA, TC, TG, Crea, FPG, smoking. To ensure that the univariate logistic regression model does not overfit, LASSO was performed to eliminate highly correlated factors, resulting in 10 variables with non-zero coefficients, including age, gender, BMI, WHR, ALT, LDL, HDL, UA, TG, smoking (Fig. 3). Next, we use multivariate logistic regression analysis to identify independent factors closely related to MAFLD. The results showed nine variables could be used as independent predictors of MAFLD (Table 2), including age, gender, BMI, WHR, ALT, LDL, HDL, UA, and smoking.

**Table 1:** Univariable logistic regression for training group.

Variables	OR	95% CI	*P*
Age	1.070	1.066–1.075	< 0.001
Sex			
Female			NA
Male	1.893	1.776–2.018	< 0.001
BMI			
< 18.5 kg/m2			NA
18.50–22.99 kg/m2	0.257	0.227–0.290	< 0.001
23.00–24.99 kg/m2	1.824	1.639–2.030	< 0.001
≥ 25.00 kg/m2	11.006	9.972–12.146	< 0.001
SBP			
SBP ≥ 130 mmHg/history of hypertension	1.585	1.490–1.685	< 0.001
< 130 mmHg			NA
DBP			
DBP ≥ 85 mmHg/history of hypertension	1.735	1.615–1.863	< 0.001
DBP < 85 mmHg			NA
WC			
Male < 90 cm/female < 80 cm			NA
Male ≥ 90 cm/female ≥ 80 cm	2.402	2.276–2.534	< 0.001
WHR			
Male < 0.90/female < 0.85			NA
Male ≥ 90 cm/female ≥ 80 cm	1.451	1.376–1.530	< 0.001
NEU	1.006	0.988–1.025	0.491
LYM	1.005	0.963–1.050	0.817
WBC	1.010	0.995–1.025	0.195
RBC	1.032	0.988–1.078	0.160
PLT	1.000	1.000–1.001	0.765
HGB	1.000	0.998–1.002	0.934
ALT	1.014	1.013–1.015	< 0.001
AST	1.013	1.011–1.015	< 0.001
ALP	1.000	0.999–1.001	0.555
LDL			
< 3.4 mmol/L			NA
≥ 3.4 mmol/L	1.980	1.870–2.098	< 0.001
HDL			
Male < 1 mmol/L/Female < 1.3 mmol/L	0.432	0.409–0.456	< 0.001
Male ≥ 1 mmol/L/Female ≥ 1.3 mmol/L			NA
UA			
≤ 420 μmol/L			NA
> 420 μmol/L	1.658	1.572–1.748	< 0.001
TC			
< 5.2 mmol/L			NA
≥ 5.2 mmol/L	1.492	1.407–1.582	< 0.001
TG			
< 1.7 mmol/L			NA
≥ 1.7 mmol/L	1.701	1.613–1.794	< 0.001
TP	1.005	0.999–1.012	0.100
GLO	0.996	0.989–1.004	0.354
ALB	1.005	0.997–1.014	0.226
TBIL	1.001	0.997–1.006	0.639
DBIL	0.997	0.982–1.012	0.686
IBIL	1.003	0.997–1.010	0.309
Crea	1.015	1.013–1.016	< 0.001
GFR	0.998	0.995–1.000	0.054
FPG			
< 5.6 mmol/L			NA
≥ 5.6 mmol/L	1.387	1.278–1.505	< 0.001
Smoke			
Yes	1.675	1.588–1.766	< 0.001
No			NA

**Table 2:** Multivariate logistic regression in training group.

Variables	OR	95% CI	*P*
Age	1.061	1.055–1.066	< 0.001
Sex			
Female			NA
Male	1.272	1.174–1.379	< 0.001
BMI			
< 18.5 kg/m^2^			NA
18.50–22.99 kg/m^2^	0.283	0.249–0.322	< 0.001
23.00–24.99 kg/m^2^	1.731	1.544–1.941	< 0.001
≥ 25.00 kg/m^2^	9.028	8.119–10.040	< 0.001
WHR			
Male < 0.90/female < 0.85			NA
Male ≥ 0.90/female ≥ 0.85	1.154	1.077–1.236	< 0.001
ALT	1.007	1.006–1.009	< 0.001
LDL			
< 3.4 mmol/L			NA
≥ 3.4 mmol/L	1.469	1.364–1.583	< 0.001
HDL			
Male < 1 mmol/L/female < 1.3 mmol/L	0.432	0.403–0.463	< 0.001
Male ≥ 1 mmol/L/female ≥ 1.3 mmol/L			NA
UA			
≤ 420 μmol/L			NA
> 420 μmol/L	1.181	1.102–1.266	< 0.001
TG			
< 1.7 mmol/L			NA
≥ 1.7 mmol/L	1.065	0.995–1.140	0.071
Smoke			
Yes	1.674	1.566–1.790	< 0.001
No			NA

### Nomogram construction

**Figure 4:**
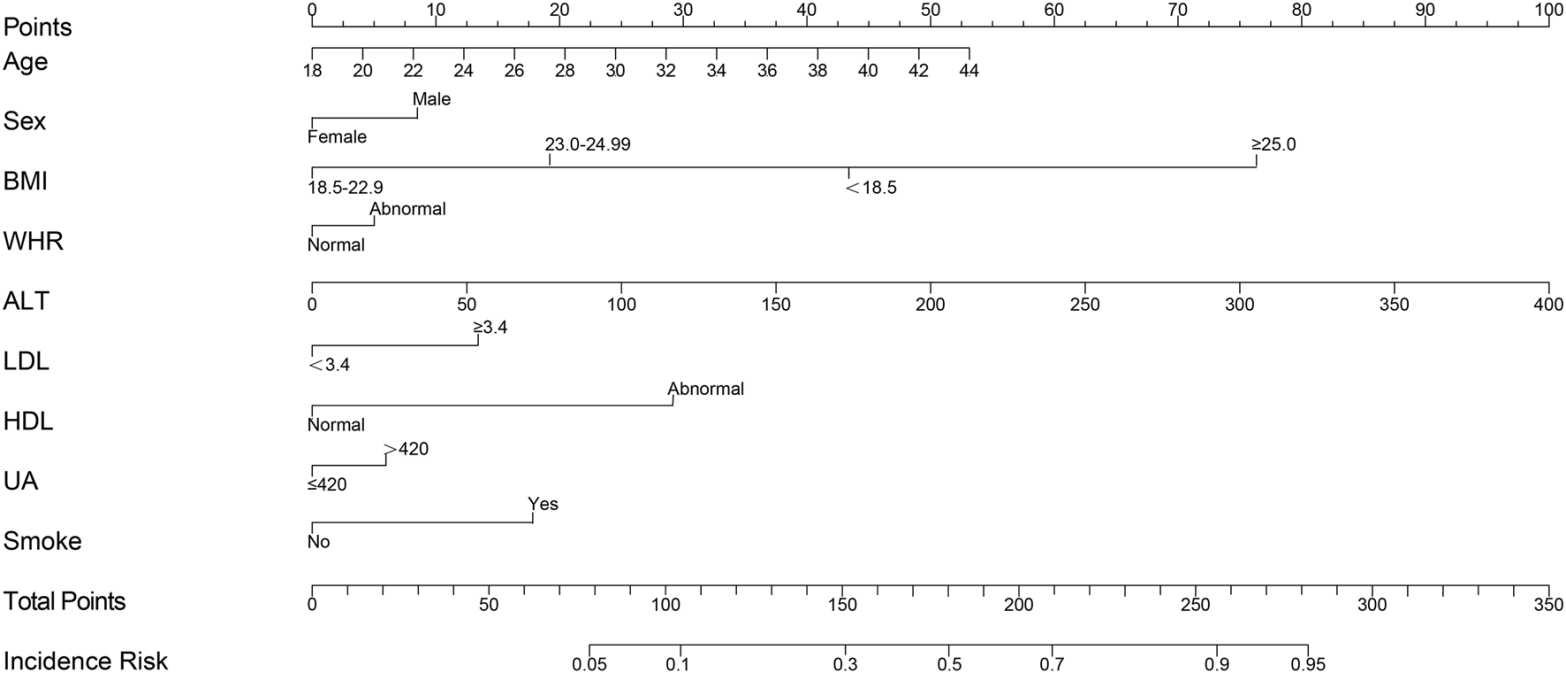
Nomogram for predicting risk of MAFLD.

A nomogram was established on the basis of the results of multivariate logistic regression analysis to predict the risk of MAFLD with age, gender, BMI, WHR, ALT, LDL, HDL, UA, and smoking (Fig. 4). A score for that variable is obtained based on the scale corresponding to the top of each predictor variable. The total score is the sum of the individual scores. When the total score corresponds vertically down the scale to the likelihood of diagnosis, the risk of each individual can be calculated.

### Verification and calibration of nomogram

ROC curves were used to assess the discriminative power of the nomogram models. The AUROC for the training and validation groups are 0.874 and 0.875, respectively (Supplementary Fig. 1).

In calibration curves, the dashed lines represent the ideal model and the solid lines represent the predictive performance of the nomogram. The closer the distance between the two lines, the better the performance of the nomogram. The overall calibration ability was good in our model (supplementary Fig. 2).

The DCA of the nomogram is shown in Supplementary Fig. 3. Decision curves showed that the model provided a more significant net gain in predicting the risk of developing MAFLD than the “all” or “none” strategy when the individual’s threshold probability was in the range of approximately 0.03–0.83.

## Discussion

Health examination has increasingly become a mean of public health management with the enhancement of health awareness. Due to the insidious onset of MAFLD, there is a lack of specific clinical symptoms and signs^16^. Therefore, MAFLD is often diagnosed incidentally during a health examination. Awareness and concern about MAFLD are generally low in the younger population. Research has found that weight loss, calorie restriction, and exercise may reverse the process of MAFLD in its early stages^17^. Accordingly, given the impact of MAFLD on health, early detection and intervention, particularly among young people, may play a key role in reducing the future national health burden.

This study based on the data from health checkups at The Affiliated Hospital of Southwest Medical University, variables were screened by logistic regression and LASSO regression analyses. We finally established a nomogram for predicting the risk of MAFLD in the younger population by using indicators that are easily accessible in various healthcare facilities. Testing of the model revealed our nomogram has good performance in terms of discrimination, calibration, and clinical utility and thus can intuitively predict the risk of MAFLD in the young population.

The model utilises easily accessible variables, such as demographic characteristics (age, sex), lifestyle habits (smoking history), anthropometrics (BMI, WHR), and laboratory data (ALT, LDL, HDL, and UA).

The correlation between demographic characteristics and MAFLD has been reported in previous studies. The risk of MAFLD increases with age. A study by Lazo M et al. showed the incidence of MAFLD is 16.1% in the 30–40 age group, 22.3% in the 41–50 age group, 29.3% in the 51–60 age group, and 27.6% in the 60 + age group^18^.

Our study also found a higher risk of MAFLD in men compared to women, which is similar to previous studies. Although the mechanism is still unclear, some researchers believe that estrogen deficiency will exacerbate liver inflammation and accelerate the progression of the disease^19^.

Regarding lifestyle, our study found smoking to be an independent predictor of MAFLD. Epidemiological evidence on the association of smoking with MAFLD has shown conflicting results in previous studies. In a cross-sectional study of 90 patients with MAFLD, no association was found between smoking and MAFLD^20^. Another cross-sectional study of 2811 participants found a 1% increased risk of MAFLD among those who smoked an additional pack of cigarettes per day^21^. Potential mechanisms underlying the association between smoking and MAFLD may be related to insulin resistance, hyperinsulinemia, dyslipidemia, hepatic steatosis, inflammation, and elevated catecholamine and glucagon levels^22^.

There is an international consensus on the relevance of anthropometry and MAFLD. Multiple studies have shown a strong relationship between obesity and MAFLD. BMI and WHR are commonly used to assess overweight or obesity, which has been included in the diagnostic criteria for MAFLD. Their risk has been confirmed in several studies^23,24^. However, recent years, studies have found that the incidence of MAFLD in the non-obese population is rising. Ye et al. showed that about 40% of patients with MAFLD are non-obese, and nearly one-fifth are lean. Both the non-obese and lean groups had substantial long-term hepatic and non-hepatic comorbidities^25^. Indications reveal that obesity should not be the only criterion for MAFLD, we should also pay enough attention to those with normal or low BMI and WHR.

In our study, four laboratory indicators were independent predictors of MAFLD, including ALT, LDL, HDL, and UA. ALT is a liver enzyme found primarily in the liver and is commonly used to indicate the presence of liver disease, which is a specific marker of liver inflammation and hepatocellular injury^26^. However, some researchers believe that a strict ALT threshold is needed to evaluate the occurrence and development of MAFLD, inaccurate ALT threshold may lead to an underestimation of liver injury^27^. Abnormal LDL and HDL levels reflect abnormalities in lipid metabolism, strongly associated with MAFLD^28^. Our study presented similar results. Souza et al. have shown that low levels of HDL-C are a risk factor for MAFLD. Low HDL-C levels may be caused by physical inactivity, obesity, and diabetes^29^. Through the cholesterol reverse transport pathway, HDL-C can promote dietary cholesterol’s efflux to exert anti-inflammatory and antioxidant effects^30^. Elevated levels of UA are commonly observed in patients with metabolic syndrome. In our study, high UA level is an independent predict factor for the risk of MAFLD, which is similar to previous studies^31^. However, some researchers argue that while previous studies have demonstrated a correlation between hyperuricemia and MAFLD, establishing a causal relationship is challenging^32^.

In summary, our study used readily available clinical and laboratory data to construct a nomogram model, which could predict the risk of MAFLD in the young Chinese population. This model suggests that MAFLD can be prevented by reducing fat, losing weight, and smoking cessation, which is significant for disease prevention and health care of young people. In addition, our nomogram model can support clinicians in screening for MAFLD and determining whether patients need further abdominal ultrasonography to confirm the diagnosis of MAFLD. Meanwhile, it can provide self-management for potentially at-risk patients with MAFLD to seek timely medical attention.

## Limitations

There are also some limitations of our study. Firstly, this study was a single-center retrospective study, which may introduce bias. Furthermore, previous studies have suggested that potential factors such as exercise, sleep, anxiety-depression scores, and dietary patterns are strongly associated with the development of MAFLD. However, we were unable to obtain this data from the medical examiners and therefore cannot provide recommendations for further lifestyle modifications. Additionally, although our sonographers have received professional training, liver biopsy remains the gold standard for the diagnosis of MAFLD. However, it is impractical to carry out liver biopsy in a large-scale medical examination population. Future studies should aim to increase the use of liver biopsy to improve the accuracy of MAFLD diagnosis.

## Conclusions

By analyzing demographic characteristics, living habits, anthropometry, and laboratory data, this study constructed a nomogram for predicting the risk of MAFLD in young people. Healthcare professionals can use this model to analyze the risk of MAFLD in patients and provide individualized prevention and treatment plans based on risk assessment. Early intervention to prevent disease progression may reduce the risk of adverse outcomes.

### Supplementary Information

Supplementary Information 1.

Supplementary Information 2.

Supplementary Information 3.

Supplementary Information 4.

## Data Availability

No datasets were generated or analysed during the current study.
