# Supplementary material for: Development and validation of a nomogram model for predicting the risk of MAFLD in the young population
Source: Sci Rep. 2024 Apr 23;14:9376. doi: 10.1038/s41598-024-60100-y (PMC11039663; doi:10.1038/s41598-024-60100-y)
Supplement: Supplementary file 1 — Supplementary Information 1. [file 41598_2024_60100_MOESM1_ESM.docx]

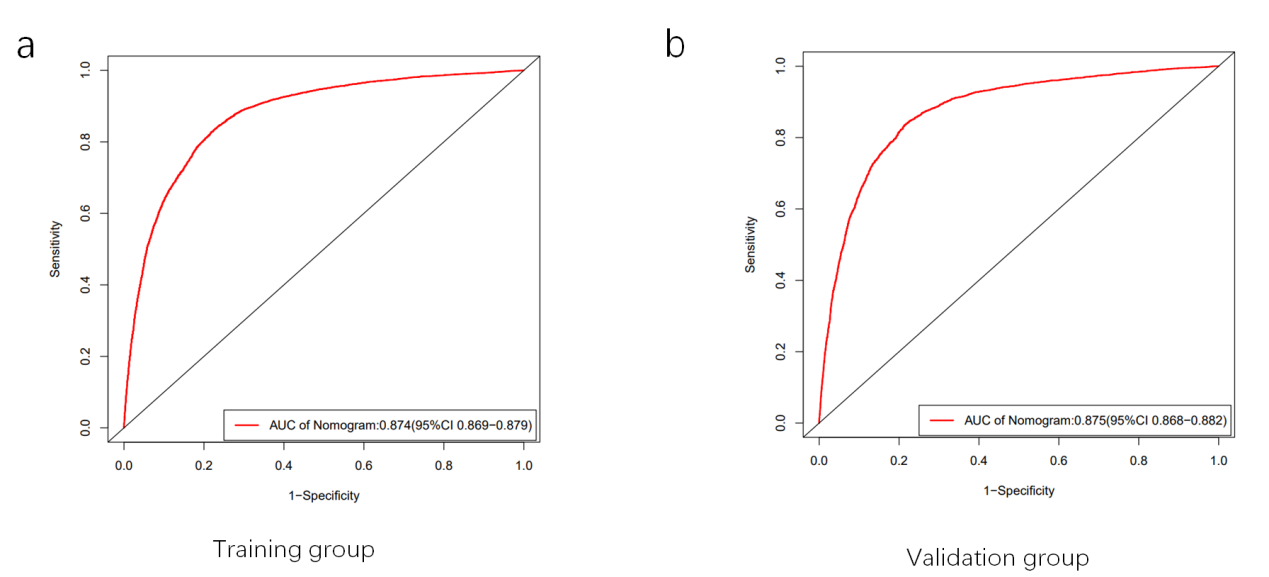


**Supplementary Fig.1** ROC Curves Validation Nomogram Discriminative Power in Training group (a) and Validation group(b)
