# Supplementary material for: Development and validation of a nomogram model for predicting the risk of MAFLD in the young population
Source: Sci Rep. 2024 Apr 23;14:9376. doi: 10.1038/s41598-024-60100-y (PMC11039663; doi:10.1038/s41598-024-60100-y)
Supplement: Supplementary file 2 — Supplementary Information 2. [file 41598_2024_60100_MOESM2_ESM.docx]

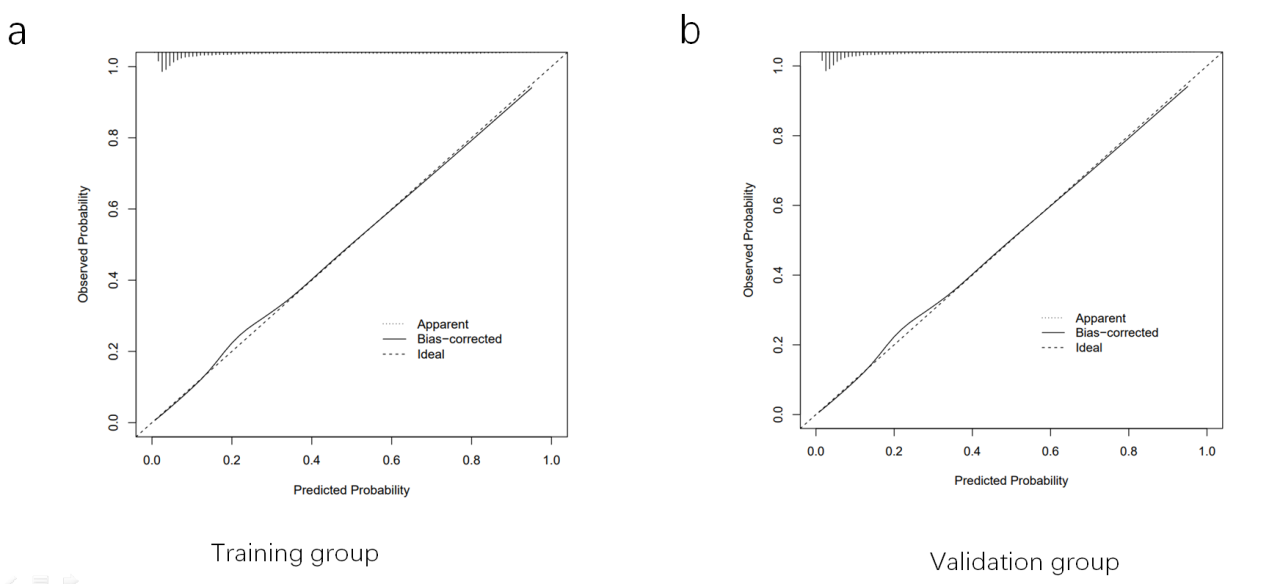


**Supplementary Fig.2** Calibration curves of the nomograms in the training group (a) and validation group (b). The dashed lines represent the ideal model and the solid lines represent the predictive performance of the nomogram. The closer the distance between the two lines, the better the performance of the nomogram.
