# Supplementary material for: Development and validation of a nomogram model for predicting the risk of MAFLD in the young population
Source: Sci Rep. 2024 Apr 23;14:9376. doi: 10.1038/s41598-024-60100-y (PMC11039663; doi:10.1038/s41598-024-60100-y)
Supplement: Supplementary file 3 — Supplementary Information 3. [file 41598_2024_60100_MOESM3_ESM.docx]

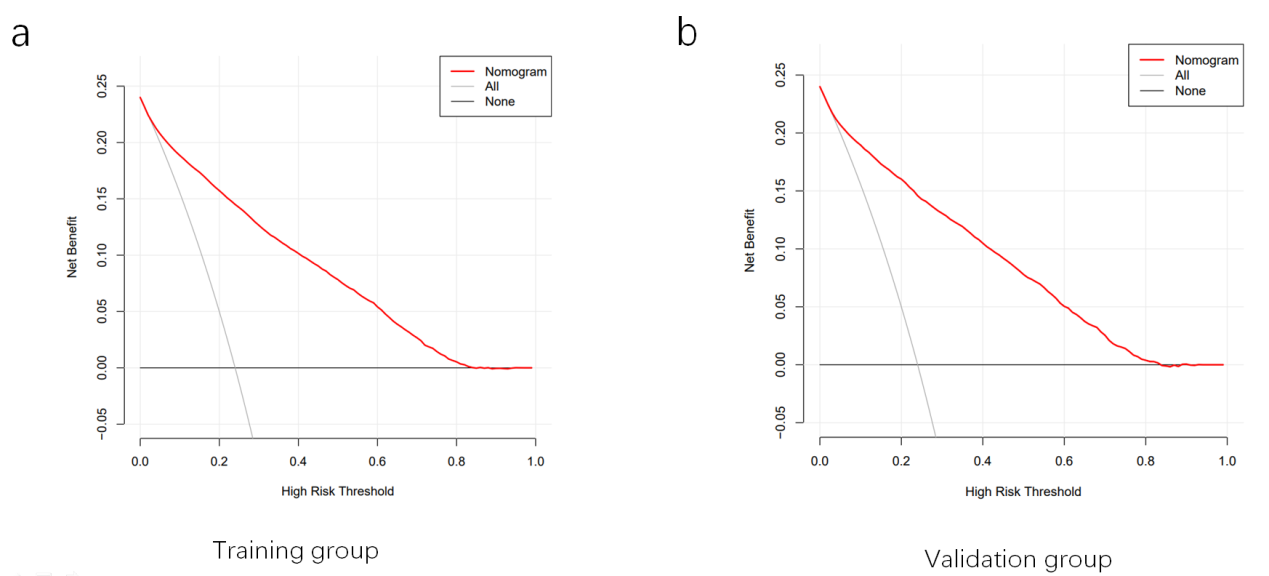


**Supplementary Fig. 3**  Clinical utility of the nomogram assessed by decision curves in the training dataset ( a ) and validation dataset ( b ). x-axis measures the threshold probability. y-axis represents the net benefit, calculated by subtracting the relative harms (false positives) from the benefits (true positives).
