# Supplementary material for: Development and validation of a nomogram model for predicting the risk of MAFLD in the young population
Source: Sci Rep. 2024 Apr 23;14:9376. doi: 10.1038/s41598-024-60100-y (PMC11039663; doi:10.1038/s41598-024-60100-y)
Supplement: Supplementary file 4 — Supplementary Information 4. [file 41598_2024_60100_MOESM4_ESM.docx]

**Supplementary table .1** Comparison of baseline features between training and validation groups

| Variables | training_group | validation_group | ***P*** |
| --- | --- | --- | --- |
| Negative | 22764 (75.6%) | 9763 (75.6%) | 0.912 |
| Positive | 7364 (24.4%) | 3149 (24.4%) | NA |
| Age | 32.82 ± 6.62 | 32.94 ± 6.6 | 0.079 |
| NEU | 3.97 ± 1.42 | 3.97 ± 1.44 | 0.471 |
| LYM | 2.15 ± 0.61 | 2.15 ± 0.61 | 0.968 |
| WBC | 6.71 ± 1.77 | 6.74 ± 1.78 | 0.249 |
| RBC | 5.08 ± 0.6 | 5.09 ± 0.6 | 0.335 |
| PLT | 237.18 ± 58.66 | 237.05 ± 57.94 | 0.879 |
| HGB | 153.73 ± 15.72 | 153.67 ± 15.62 | 0.442 |
| ALT | 35.54 ± 22.92 | 35.39 ± 22.77 | 0.505 |
| AST | 26.52 ± 13.21 | 26.43 ± 13.27 | 0.501 |
| ALP | 72.01 ± 23.42 | 71.94 ± 23 | 0.987 |
| TP | 73.51 ± 4.21 | 73.5 ± 4.22 | 0.910 |
| GLO | 26.94 ± 3.46 | 26.92 ± 3.45 | 0.719 |
| ALB | 47.08 ± 3.14 | 47.05 ± 3.14 | 0.430 |
| TBIL | 14.98 ± 5.71 | 15.07 ± 5.77 | 0.219 |
| DBIL | 4.63 ± 1.78 | 4.65 ± 1.71 | 0.219 |
| IBIL | 10.34 ± 4.15 | 10.41 ± 4.14 | 0.076 |
| Crea | 66.72 ± 16.45 | 66.74 ± 19.08 | 0.651 |
| GFR | 123.89 ± 11.19 | 123.79 ± 11.23 | 0.337 |
| Female | 8724 (29%) | 3700 (28.7%) | 0.531 |
| Male | 21404 (71%) | 9212 (71.3%) | NA |
| SBP＜130mmHg | 23952 (79.5%) | 10245 (79.3%) | 0.716 |
| SBP≥130mmHg/History of  hypertension | 6176 (20.5%) | 2667 (20.7%) | NA |
| DBP＜85mmHg | 26086 (86.6%) | 11235 (87%) | 0.233 |
| DBP≥85mmHg/History of  hypertension | 4042 (13.4%) | 1677 (13%) | NA |
| WC Male＜90cm/Female＜80cm | 19661 (65.3%) | 8401 (65.1%) | 0.699 |
| WC Male≥90cm/Female≥80cm | 10467 (34.7%) | 4511 (34.9%) | NA |
| WHR Male＜0.90/Female＜0.85 | 15440（51.2%） | 6621（51.3%） | 0.958 |
| WHR Male≥0.90/Female≥0.85 | 14688（48.8%） | 6291（48.7%） | NA |
| LDL＜3.4mmol/L | 22761 (75.5%) | 9759 (75.6%) | 0.951 |
| LDL≥3.4mmol/L | 7367 (24.5%) | 3153 (24.4%) | NA |
| HDL Male <1mmol/L/Female <1.3mmol/L | 10178 (33.8%) | 4314 (33.4%) | 0.456 |
| HDL Male ≥1mmol/L/Female ≥1.3mmol/L | 19950 (66.2%) | 8598 (66.6%) | NA |
| UA ≤420μmol/L | 18661 (61.9%) | 7969 (61.7%) | 0.665 |
| UA >420μmol/L | 11467 (38.1%) | 4943 (38.3%) | NA |
| TC＜5.2mmol/L | 22764 (75.6%) | 9713 (75.2%) | 0.464 |
| TC≥5.2mmol/L | 7364 (24.4%) | 3199 (24.8%) | NA |
| TG＜1.7mmol/L | 16200 (53.8%) | 7054 (54.6%) | 0.102 |
| TG≥1.7mmol/L | 13928 (46.2%) | 5858 (45.4%) | NA |
| FPG＜5.6mmol/L | 27032 (89.7%) | 11584 (89.7%) | 0.987 |
| FPG≥5.6mmol/L | 3096 (10.3%) | 1328 (10.3%) | NA |
| No smoke | 15801 (52.4%) | 6828 (52.9%) | 0.411 |
| Smoke | 14327 (47.6%) | 6084 (47.1%) | NA |
| BMI <18.5Kg/㎡ | 4181 (13.9%) | 1775 (13.7%) | 0.679 |
| BMI 18.50–22.99Kg/㎡ | 12729 (42.2%) | 5527 (42.8%) | NA |
| BMI 23.00–24.99Kg/㎡ | 5521 (18.3%) | 2316 (17.9%) | NA |
| BMI ≥25.00Kg/㎡ | 7697 (25.5%) | 3294 (25.5%) | NA |
